# Supplementary material for: The Impact of the COVID-19 Pandemic on Influenza Vaccination Attitudes and Actions in Spain’s Adult Population
Source: Vaccines (Basel). 2023 Sep 23;11(10):1514. doi: 10.3390/vaccines11101514 (PMC10611015; doi:10.3390/vaccines11101514)
Supplement: Supplementary file 1 [file vaccines-11-01514-s001.zip › Questionnaire - 2022 - EN.pdf]

Good morning/afternoon, my name is... I am calling from GAD3. We are conducting a survey to find out what citizens think about influenza vaccination. The survey is anonymous and takes 4 minutes, thank you very much for your cooperation!

Sub-sample 1

Sub-sample 2

Profile

Q01 Sex

Man	1
Woman	2

Q02 Age

Introduction

Q03 To begin with, on a health level, could you mention any of the so-called risk groups that are susceptible to flu vaccination? (SPONTANEOUS. Do not read.)

	Get it right	Not right
People over 65 years of age	1	2
Children under 2 years of age	1	2
Diabetics	1	2
Cardiac pathologies	1	2
Respiratory pathologies	1	2
Immunosuppression	1	2
Other chronic or immunosuppressed pathologies	1	2
Health professionals	1	2
Pregnant women	1	2
People living with at-risk patients	1	2
People with disabilities	1	2
Essential workers	1	2
State security forces	1	2

Q04 Do you belong to any risk group?

Yes	1	
No	2	Go to Q06
NS/NC	3	

Q05 What type of group? MULTI-RESPONSE

	Yes	No
People over 65 years of age	1	2
Diabetics	1	2
Cardiac pathologies	1	2
Respiratory pathologies	1	2
Immunosuppression	1	2
Other chronic or immunosuppressed pathologies	1	2

Health professionals	1	2
Pregnant women	1	2
People living with at-risk patients	1	2
People with disabilities	1	2
Essential workers	1	2
State security forces	1	2
Another	1	2

Q06 In the last few years, have you ever had a flu vaccination?

Yes, annually	1	
Yes, with some frequency	2	
Yes, sporadically	3	
No	4	Go to Q8
NS/NC	5	

Q07 Which of the following reasons did you consider most relevant to your decision to be vaccinated? (Please tick no more than 3 response options)

	Yes	No	NS/NC
For my own protection and/or that of my environment	1	2	3
Because I have sufficient information about the vaccine	1	2	3
Because I have previously suffered the consequences of influenza	1	2	3
Because a doctor recommended it	1	2	3
Because it was recommended to me by nurses	1	2	3
For social responsibility	1	2	3
To get both flu and Covid vaccinations	1	2	3

GO TO Q9

Q08 Of the following reasons, which did you consider most relevant to your decision NOT to be vaccinated? (Please tick no more than 3 answer options) (IF Q06=4 or 5)

	Yes	No	NS/NC
Lack of confidence in vaccine effectiveness	1	2	3
I do not consider the flu to be serious.	1	2	3
I do not have enough information about the vaccine.	1	2	3
I have a phobia of needles	1	2	3
I was previously vaccinated and it made me sick.	1	2	3
I have not been prescribed it	1	2	3

Knowledge and appreciation of the vaccine

Q09 Over the last year, have you received any information about the flu vaccination campaign?

Yes	1	
No	2	Go to Q11
NS/NC	3	

Q10 And through what medium did you receive this information?

Public administration	1

Health personnel (general practitioners, other doctors, nurses, etc.)	2
Media (TV, radio, press)	3
Social media	4
Family or friends	5
In my workplace	6
For myself	7
Another	8
NS/NC	9

Q11 And to what extent do you think the following actions may encourage influenza vaccination this year?

	A lot	Quite	Little	Nothing	NS/NC
That people around me decide to get vaccinated	1	2	3	4	5
A media awareness campaign	1	2	3	4	5
Facilitating access to Primary Care Centres	1	2	3	4	5
Creation of "vaccinodromes" like those of Covid	1	2	3	4	5
Sending annual reminders via SMS	1	2	3	4	5
Learn more about vaccination and its benefits	1	2	3	4	5

Influenza vaccine

Q12 And, in view of the upcoming flu vaccination campaign, do you intend to get vaccinated?

Yes	1	
No	2	Go to Q14
NS/NC	3	

Q13 Which of the following reasons is most relevant to your decision to be vaccinated? (Please tick no more than 3 answer options)

	Yes	No	NS/NC
Protection against viruses is necessary	1	2	3
Flu vaccinations are necessary every year	1	2	3
I trust vaccines in general	1	2	3
For social responsibility	1	2	3
Because Covid has made me more aware of the importance of vaccination.	1	2	3
To get both flu and Covid vaccinations	1	2	3

GO TO Q15

Q14 Of the following reasons, which are the most relevant for deciding NOT to be vaccinated? (Please tick 3 answer options maximum)

	Yes	No	NS/NC
I consider that flu is not a serious virus (it is not fatal).	1	2	3
COVID-19 vaccine is sufficient.	1	2	3
No vaccination required	1	2	3
I don't trust vaccines in general	1	2	3
Not effective	1	2	3
Not recommended/prescribed	1	2	3

Q15 At which location would you like to go for a flu vaccination? MULTI-COUNTRY Select maximum 2

	Yes	No	NS/NC
Health centre	1	2	3
Hospital	1	2	3
Specific vaccination zone (Covid type)	1	2	3
Workplace	1	2	3
Home delivery, in case of mobility problems	1	2	3
Another	1	2	3

Q16 In your opinion, do you think that in the context of the Covid pandemic, the flu vaccine is more or less important than in other years?

More importantly	1
Equally important (do not read)	2
Less important	3
NS/NC	4

Q17 And, given the vulnerability of the elderly, do you consider it necessary for the administration to purchase a specific type of influenza vaccine for this population?

Yes, in any case	1
Yes, but it depends on the price	2
It is not necessary to	3
NS/NC	4

Profile

Q18 Finally, what is your current employment situation?

Private sector worker	1
Public sector worker	2
Self-employed/Entrepreneur	3
Retired	4
Unemployed	5
Student	6
Domestic worker	7

Q19 Could you please tell me your level of education?

Primary or basic	1
Secondary	2
University students	3

Q20 Please indicate your postal code of residence:

Thank you very much for your cooperation
