# Supplementary material for: The Impact of the COVID-19 Pandemic on Influenza Vaccination Attitudes and Actions in Spain’s Adult Population
Source: Vaccines (Basel). 2023 Sep 23;11(10):1514. doi: 10.3390/vaccines11101514 (PMC10611015; doi:10.3390/vaccines11101514)
Supplement: Supplementary file 1 [file vaccines-11-01514-s001.zip › Questionnaire - 2022 - ES.pdf]

Buenos días/tardes, mi nombre es… le llamo de GAD3. Estamos realizando un estudio para conocer la opinión de los ciudadanos sobre la vacunación frente a la gripe. La encuesta es anónima y dura 4 minutos. ¡Muchas gracias por su colaboración!

Submuestra 1

Submuestra 2

Perfil

Q01 Sexo

Hombre	1
Mujer	2

Q02 Edad

Introducción

Q03 Para empezar, a nivel sanitario, ¿sabría mencionar alguno de los denominados grupos de riesgo susceptibles de ser vacunados de gripe? (ESPONTÁNEA. No leer.)

	Acierta	No acierta
Personas mayores de 65 años	1	2
Niños menores de 2 años	1	2
Diabéticos	1	2
Patologías cardíacas	1	2
Patologías respiratorias	1	2
Inmunosupresión	1	2
Otras patologías crónicas o inmunodeprimidas	1	2
Profesionales sanitarios	1	2
Mujeres embarazadas	1	2
Personas convivientes con pacientes de riesgo	1	2
Personas con discapacidad	1	2
Trabajadores esenciales	1	2
Cuerpos y fuerzas de seguridad del Estado	1	2

Q04 ¿Pertenece Ud. a algún grupo de riesgo?

Sí	1	
No	2	Pasar a Q06
NS/NC	3	

Q05 ¿A qué tipo de grupo? MULTIRRESPUESTA

	Sí	No
Personas mayores de 65 años	1	2
Diabéticos	1	2
Patologías cardíacas	1	2
Patologías respiratorias	1	2
Inmunosupresión	1	2
Otras patologías crónicas o inmunodeprimidas	1	2
Profesionales sanitarios	1	2
Mujeres embarazadas	1	2

Personas convivientes con pacientes de riesgo	1	2
Personas con discapacidad	1	2
Trabajadores esenciales	1	2
Cuerpos y fuerzas de seguridad del Estado	1	2
Otro	1	2

Q06 En los últimos años, ¿se ha vacunado Ud. de la gripe en alguna ocasión?

Sí, anualmente	1	
Sí, con cierta frecuencia	2	
Sí, esporádicamente	3	
No	4	Pasa a Q8
NS/NC	5	

Q07 De los siguientes motivos, ¿cuáles consideró más relevantes de cara a decidir vacunarse? (Señalar máximo 3 opciones de respuesta)

	Sí	No	NS/NC
Por mi propia protección y/o de mi entorno	1	2	3
Porque dispongo de suficiente información sobre la vacuna	1	2	3
Porque he sufrido anteriormente las consecuencias de la gripe	1	2	3
Porque me lo recomendó un médico	1	2	3
Porque me lo recomendaron enfermeros	1	2	3
Por responsabilidad social	1	2	3
Para vacunarme a la vez contra la gripe y contra el Covid	1	2	3

PASAR A Q9

Q08 De los siguientes motivos, ¿cuáles consideró más relevantes de cara a decidir NO vacunarse? (Señalar máximo 3 opciones de respuesta) (SI Q06=4 o 5)

	Sí	No	NS/NC
Falta de confianza en la efectividad de la vacuna	1	2	3
Considero que la gripe no reviste gravedad	1	2	3
No dispongo de suficiente información sobre la vacuna	1	2	3
Tengo fobia a las agujas	1	2	3
Me vacuné previamente y me sentó mal	1	2	3
No me lo han prescrito	1	2	3

Conocimiento y valoración de la vacuna

Q09 A lo largo del último año, ¿ha recibido algún tipo de información sobre la campaña de vacunación de la gripe?

Sí	1	
No	2	Pasa a Q11
NS/NC	3	

Q10 Y, ¿a través de qué medio ha recibido dicha información?

Administración pública	1
Personal sanitario (médico de cabecera, otros médicos, enfermeros, etc)	2

Medios de comunicación (TV, radio, prensa)	3
Redes sociales	4
Familiares o amigos	5
En mi centro de trabajo	6
Por mí mismo	7
Otro	8
NS/NC	9

Q11 ¿Y en qué medida considera Ud. que las siguientes acciones pueden incentivar la vacunación de la gripe este año?

	Mucho	Bastante	Poco	Nada	NS/NC
Que las personas de mi entorno decidan vacunarse	1	2	3	4	5
Una campaña de concienciación en medios	1	2	3	4	5
Facilitar el acceso a los Centros de Atención Primaria	1	2	3	4	5
Creación de "vacunódromos" como los del Covid	1	2	3	4	5
Envío de recordatorios anuales vía SMS	1	2	3	4	5
Tener más información sobre la vacunación y sus beneficios	1	2	3	4	5

Vacuna de la gripe

Q12 Y, de cara a la próxima campaña de vacunación sobre la gripe, ¿tiene intención de vacunarse?

Sí	1	
No	2	Pasar a Q14
NS/NC	3	

Q13 De los siguientes motivos, ¿cuál es más relevantes de cara a decidir vacunarse? (Señalar máximo 3 opciones de respuesta)

	Sí	No	NS/NC
Es necesario protegerse frente a los virus	1	2	3
Es necesario vacunarse cada año de gripe	1	2	3
Confío en las vacunas en general	1	2	3
Por responsabilidad social	1	2	3
Porque el Covid me ha hecho ser más consciente de la importancia de la vacunación	1	2	3
Para vacunarme a la vez contra la gripe y contra el Covid	1	2	3

PASAR A Q15

Q14 De los siguientes motivos, ¿cuáles son los más relevantes de cara a decidir NO vacunarse? (Señalar máximo 3 opciones de respuesta)

	Sí	No	NS/NC
Considero que la gripe no es un virus grave (no es mortal)	1	2	3
Es suficiente con la vacuna del COVID-19	1	2	3
No es necesario vacunarse	1	2	3
No confío en las vacunas en general	1	2	3
No es efectiva	1	2	3
No me lo han recomendado/ prescrito	1	2	3

Q15 ¿En qué ubicación le gustaría poder acudir a vacunarse contra la gripe? MULTIRRESPUESTA
Seleccionar máximo 2

	Sí	No	NS/NC
Centro de salud	1	2	3
Hospital	1	2	3
Zona específica de vacunación (tipo Covid)	1	2	3
Centro de trabajo	1	2	3
A domicilio, en caso de tener problemas de movilidad	1	2	3
Otro	1	2	3

Q16 En su opinión, ¿cree que en el contexto de pandemia de Covid, la vacuna de la gripe es más o menos importante que otros años?

Más importante	1
Igual de importante (no leer)	2
Menos importante	3
NS/NC	4

Q17 Y, teniendo en cuenta la vulnerabilidad de las personas mayores, ¿considera necesario que la administración adquiera un tipo específico de la vacuna contra la gripe para esta población?

Sí, en cualquier caso	1
Sí, pero depende del precio	2
No es necesario	3
NS/NC	4

Perfil

Q18 Para terminar, ¿cuál es su situación laboral actual?

Trabajador del sector privado	1
Trabajador del sector público	2
Autónomo/Empresario	3
Jubilado	4
Desempleado	5
Estudiante	6
Trabajador domestico	7

Q19 ¿Podría indicarme cuál es su nivel de estudios?

Primarios o básicos	1
Secundarios	2
Universitarios	3

Q20 Indique su código postal de residencia:

Muchas gracias por su colaboración
